# From Awareness to Action: A Review of Efforts to Reduce Disparities in Breast Cancer Screening

**DOI:** 10.7759/cureus.40674

**Published:** 2023-06-20

**Authors:** Shiven Nayyar, Swarupa Chakole, Avinash B Taksande, Roshan Prasad, Pratiksha K Munjewar, Mayur B Wanjari

**Affiliations:** 1 Medicine, Jawaharlal Nehru Medical College, Datta Meghe Institute of Higher Education and Research, Wardha, IND; 2 Community Medicine, Jawaharlal Nehru Medical College, Datta Meghe Institute of Higher Education and Research, Wardha, IND; 3 Physiology, Jawaharlal Nehru Medical College, Datta Meghe Institute of Higher Education and Research, Wardha, IND; 4 Internal Medicine, Jawaharlal Nehru Medical College, Datta Meghe Institute of Higher Education and Research, Wardha, IND; 5 Medical-Surgical Nursing, Srimati Radhikabai Meghe Memorial College of Nursing, Datta Meghe Institute of Higher Education and Research, Wardha, IND; 6 Research and Development, Jawaharlal Nehru Medical College, Datta Meghe Institute of Higher Education and Research, Wardha, IND

**Keywords:** patient navigation, policy, interventions, action, awareness, disparities, breast cancer screening

## Abstract

Breast cancer is a significant public health concern, and addressing disparities in breast cancer screening is crucial for improving early detection and reducing mortality rates. This review article examines efforts to bridge the gap between awareness and action in reducing disparities in breast cancer screening. A systematic approach was employed to gather relevant literature using various databases. The selected studies encompassed a range of interventions, including policy changes, community-based programs, culturally competent interventions, technological advancements, and patient navigation. The review highlights the importance of policies and legislation in improving access to screening services and the impact of community-based initiatives in addressing disparities. Culturally competent interventions, tailored messaging, and language support were found to be effective in improving screening rates among diverse populations. Technological advancements, such as telemedicine and mobile health applications, were identified as promising approaches to increase access to screening services. Patient navigation programs effectively addressed barriers to screening and improved screening rates. The review also discusses evaluating efforts, limitations, and the need for continuous improvement. Future directions and recommendations include addressing gaps in the existing literature, proposing research directions, and providing recommendations for policymakers, healthcare providers, and researchers. By implementing these recommendations and working collaboratively, we can strive for equitable access to breast cancer screening for all populations, ultimately leading to improved outcomes and reduced disparities.

## Introduction and background

Breast cancer is a significant global health concern, affecting millions of individuals worldwide. Early detection through regular breast cancer screening is crucial in reducing mortality rates and improving treatment outcomes. However, despite effective screening methods, substantial disparities exist in accessing these services among different populations. This review article aims to provide an in-depth analysis of efforts to reduce disparities in breast cancer screening, moving beyond mere awareness campaigns toward actionable strategies [1,2].

Breast cancer is the most commonly diagnosed cancer among women globally, and its incidence is increasing. The introduction of mammography and other screening modalities has revolutionized the early detection and treatment of breast cancer, improving survival rates. Early detection allows for timely intervention, potentially reducing the need for aggressive treatments and improving the overall prognosis. Therefore, promoting breast cancer screening is paramount in public health [3,4].

Despite the importance of breast cancer screening, various factors contribute to disparities in the access and utilization of these services. Socioeconomic status, race, ethnicity, geographical location, cultural beliefs, and language barriers are key determinants of these disparities. Disadvantaged populations, including low-income individuals, racial and ethnic minorities, and those residing in underserved areas, often face barriers, such as limited access to healthcare facilities, lack of health insurance, and cultural misconceptions surrounding screening [5,6].

Raising awareness about breast cancer and the importance of screening is an essential first step, but it is not sufficient. Bridging the gap between awareness and action is crucial to ensure that individuals understand the significance of screening and have access to and utilize these services. Simply disseminating information without addressing the underlying barriers and implementing targeted interventions is unlikely to reduce disparities significantly [7,8].

The primary objective of this review article is to critically examine the efforts undertaken to reduce disparities in breast cancer screening. By analyzing various strategies, programs, and policies implemented at different levels, this review aims to provide insights into the effectiveness of these interventions in promoting equitable access to screening services. In addition, the article will identify gaps in the existing literature and propose future research directions to further address disparities in breast cancer screening.

## Review

Methodology

**Figure 1:**
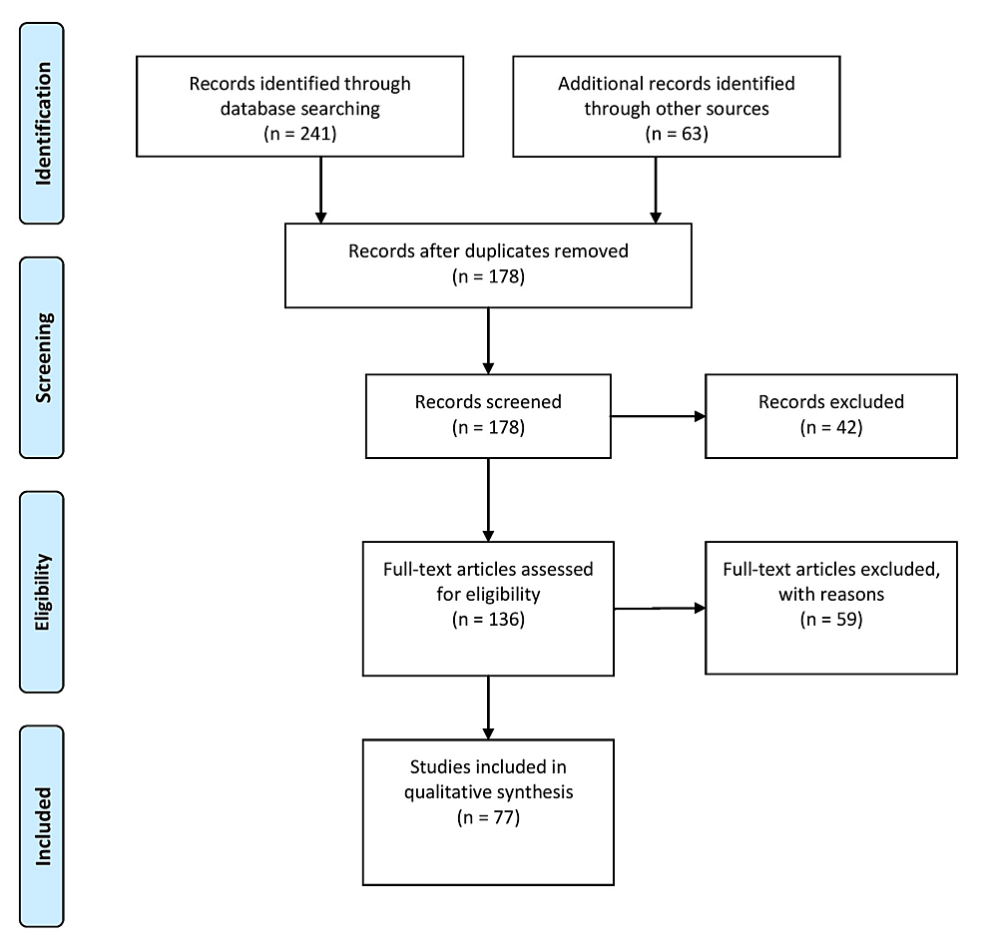
Selection process of articles used in this study. Adopted from the Preferred Reporting Items for Systematic Reviews and Meta-Analyses (PRISMA)

A systematic approach was employed in conducting this review article to gather relevant literature and provide a comprehensive analysis of efforts to reduce disparities in breast cancer screening. The literature search was conducted using databases, such as PubMed, Scopus, and Google Scholar, utilizing combinations of keywords related to breast cancer screening, disparities, interventions, awareness, and action. The search was limited to articles published in English within a specific time frame. Studies were included based on their relevance to the topic and contribution to understanding efforts to reduce disparities in breast cancer screening. Various interventions were considered, including policy changes, community-based programs, culturally competent interventions, technological advancements, and patient navigation. Figure 1 describes the selection process of articles used in our study.

Overview of policy changes and legislation aimed at reducing disparities

Policy changes and legislation have played a vital role in addressing disparities in breast cancer screening. Governments and healthcare systems worldwide have implemented various measures to ensure equitable access to screening services. These initiatives aim to eliminate barriers, improve healthcare infrastructure, and promote equal opportunities for underserved populations [3,9,10]. Legislative actions have included developing and implementing guidelines and laws prioritizing breast cancer screening and addressing disparities. For instance, some countries have mandated insurance coverage for mammography and other screening modalities, making these services more accessible and affordable for all individuals. In addition, legislative efforts have focused on expanding Medicaid or providing subsidies to ensure that low-income individuals can access screening services [1,6,7,11].

Impact of Guidelines and Laws on Access to Screening Services

Implementing guidelines and laws has substantially impacted access to breast cancer screening services. By setting clear recommendations and standards, guidelines provide healthcare providers with a framework for delivering high-quality screening services. They also facilitate consistent and evidence-based decision-making regarding the frequency, modality, and initiation of screening [7,12].

Moreover, laws mandating insurance coverage for screening services have helped reduce financial barriers and increase screening rates. Individuals who previously faced challenges due to the cost of screening can now access these services without incurring significant out-of-pocket expenses. As a result, previously underserved populations can now undergo regular breast cancer screening, leading to earlier detection and improved outcomes [7,13-15].

Successful Policy Interventions

Several successful policy interventions have been implemented to reduce disparities in breast cancer screening. For instance, some countries have established comprehensive breast cancer control programs encompassing screening, diagnosis, treatment, and survivorship. These programs emphasize the importance of early detection and provide targeted interventions to underserved populations, including educational campaigns, mobile screening units, and collaborations with community organizations [16,17].

In addition, policy initiatives have focused on addressing geographical disparities by establishing screening facilities in underserved areas or implementing mobile mammography programs to reach rural and remote communities. Such interventions aim to overcome distance barriers and lack of access to healthcare facilities, ensuring that individuals in these areas receive timely screening services [18-20]. Furthermore, policies promoting culturally sensitive care and language assistance have effectively improved screening rates among racial and ethnic minority populations. By recognizing and addressing cultural beliefs, language barriers, and mistrust in healthcare systems, these policies facilitate better engagement and uptake of screening services among diverse communities [21,22].

Community-based programs

Importance of Community-Based Initiatives in Addressing Disparities

Community-based initiatives are crucial in reducing disparities in breast cancer screening by addressing the unique challenges and barriers underserved populations face. These programs recognize the importance of engaging with communities directly and tailoring interventions to meet their needs. By focusing on the local context, community-based initiatives can foster trust, enhance awareness, and increase the uptake of screening services [23,24].

Partnerships Among Healthcare Providers, Community Organizations, and Outreach Programs

Successful community-based programs often involve partnerships among healthcare providers, community organizations, and outreach programs. Collaboration among these stakeholders allows for a comprehensive approach to address multiple barriers. Healthcare providers bring clinical expertise, while community organizations possess knowledge of the community's needs and cultural sensitivities. Outreach programs facilitate access and support throughout the screening process [9,25].

These partnerships can design and implement targeted interventions that effectively reach underserved populations by working together. They can leverage community networks, establish trusted relationships, and deliver culturally appropriate messaging to improve awareness, education, and access to screening services.

Strategies to Improve Awareness, Education, and Access to Screening Services

Community-based programs employ various strategies to improve awareness, education, and access to breast cancer screening services. Some common strategies include the following:

Community Outreach and Education: These programs organize educational workshops, community events, and awareness campaigns to disseminate information about the importance of breast cancer screening. They utilize culturally sensitive materials and employ local influencers and trusted community members as messengers [26,27].

Peer Education and Support: Peer education programs train community members who have experienced breast cancer or have undergone screening to serve as advocates and provide support. Peers can effectively address fears, misconceptions, and cultural barriers, encouraging others to seek screening [28,29].

Mobile and Mammography Vans: Mobile screening units or mammography vans are deployed to reach underserved areas, including rural and remote communities. These units offer on-site screening services, eliminating the need for individuals to travel long distances to access healthcare facilities [30,31].

Navigation and Assistance Programs: Navigation programs provide personalized assistance to individuals throughout the screening process, helping them overcome logistical and administrative barriers. Navigators offer guidance, support, and care coordination, ensuring that individuals receive timely screenings and follow-up care [32,33].

Collaborations with Faith-Based Organizations and Community Leaders: Engaging faith-based organizations and community leaders can facilitate trust building and allow for the dissemination of information within communities. By partnering with influential community figures, programs can promote screening messages and facilitate access to services [34]. These strategies empower communities, address cultural and linguistic barriers, and ensure that underserved populations have the knowledge, resources, and support necessary for breast cancer screening. By adopting a community-based approach, these programs can potentially reduce disparities and improve outcomes for individuals facing significant barriers to healthcare services.

Culturally competent interventions

Cultural Barriers and Their Impact on Screening Disparities

Cultural factors can significantly impact disparities in breast cancer screening. Cultural beliefs, language barriers, religious or traditional practices, and mistrust in healthcare systems can create obstacles to accessing and utilizing screening services. Acknowledging and addressing these cultural barriers is crucial to ensure that individuals from diverse populations can benefit from breast cancer screening [35].

Tailored Messaging and Language Support for Diverse Populations

Culturally competent interventions focus on tailoring messaging and providing language support to engage diverse populations effectively. These interventions recognize the importance of language, cultural norms, and beliefs in influencing health behaviors and decision-making. By adapting communication strategies, materials, and approaches, culturally competent interventions can overcome cultural barriers and promote the importance of breast cancer screening [36].

Tailored messaging involves using culturally appropriate language, imagery, and examples that resonate with specific communities. It considers the unique cultural contexts, values, and preferences of the target population, enhancing the relevance and effectiveness of the messaging. This approach can dispel myths, address fears, and increase awareness about breast cancer screening within diverse communities [37].

Language support is essential for individuals who have limited English proficiency. Culturally competent interventions ensure that language barriers do not hinder access to screening services. This may involve providing interpreters, multilingual educational materials, and language assistance services at healthcare facilities or screening sites. Language support helps individuals understand the importance of screening, the process involved, and the available resources [21].

Successful Culturally Competent Interventions

Numerous successful examples of culturally competent interventions have been implemented to reduce disparities in breast cancer screening:

Community Health Workers (CHWs): CHWs, who are members of the communities they serve, play a crucial role in culturally competent interventions. They deeply understand the population's cultural norms, beliefs, and languages. CHWs can effectively deliver health education, provide navigation support, and bridge the gap between healthcare systems and underserved communities [38].

Culturally Tailored Educational Materials: Developing culturally tailored educational materials involves creating materials that reflect specific communities' language, imagery, and cultural norms. These materials address cultural beliefs, myths, and misconceptions surrounding breast cancer and screening. By resonating with the target population, these materials increase awareness and encourage screening uptake [39].

Community-Based Workshops and Support Groups: Conducting community-based workshops and support groups tailored to specific cultural groups fosters a safe and culturally sensitive environment. These initiatives allow individuals to openly discuss their concerns, share experiences, and seek guidance. Peer support from individuals with similar cultural backgrounds can enhance comfort and encourage participation in screening [40].

Faith-Based Interventions: Engaging faith-based organizations, such as churches and mosques, can effectively promote breast cancer screening within religious communities. Collaborating with faith leaders and integrating screening messages into religious events or sermons can help overcome cultural and religious barriers and emphasize the importance of early detection [41].

Technology and innovation

Advancements in Technology and Their Role in Reducing Disparities

Advancements in technology have played a significant role in reducing disparities in breast cancer screening by increasing access and overcoming logistical barriers. Technological innovations can potentially reach underserved populations, improve communication, and enhance the delivery of screening services. These advancements offer new opportunities to bridge gaps and promote equitable access to breast cancer screening [42].

Telemedicine and Mobile Health Applications for Increased Access

Telemedicine and mobile health applications have emerged as powerful tools for increasing access to breast cancer screening, particularly in underserved areas. Telemedicine allows individuals to connect remotely with healthcare providers for consultations, referrals, and screening recommendations. It eliminates the need for in-person visits, reducing travel time, costs, and logistical challenges [43].

Mobile health applications (apps) provide a convenient platform for individuals to access screening information, schedule appointments, and receive reminders. These apps can also offer educational materials, risk assessment tools, and guidance on self-examinations. Mobile health apps have the potential to empower individuals to take control of their breast health and facilitate timely screening [44,45].

Furthermore, telemedicine and mobile health applications can be especially beneficial for individuals with limited mobility, those residing in rural or remote areas, and populations facing transportation barriers. By leveraging technology, these interventions improve access to screening services, increase convenience, and enhance engagement.

Successful Technological Interventions

Mobile Mammography Units: Mobile mammography units equipped with advanced imaging technology have been deployed to reach underserved populations. These units can travel to remote or rural areas, workplaces, community centers, and other locations, providing on-site screening services. Mobile mammography units have successfully increased access and screening rates in populations with limited healthcare infrastructure [18,19,46].

Telehealth Consultations: Telehealth consultations enable individuals to connect with healthcare providers remotely. Through video calls or secure online platforms, healthcare professionals can provide guidance, answer questions, and recommend appropriate screening options. Telehealth consultations improve access to expert opinions, particularly for individuals residing in areas with a shortage of healthcare providers or specialists [47].

Reminders and Educational Apps: Mobile and text message reminders have effectively increased screening rates. These apps offer educational materials, appointment reminders, and self-examination guidance. They can also track screening histories and send alerts for follow-up appointments. By leveraging smartphone technology, these interventions promote engagement and facilitate timely screening [48].

Digital Risk Assessment Tools: Online risk assessment tools allow individuals to assess their risk for breast cancer and understand their screening needs. These tools use algorithms to evaluate personal and family history, lifestyle factors, and other relevant information. Digital risk assessment tools provide tailored recommendations for screening frequency and can encourage individuals to seek appropriate screening based on their level of risk [49,50].

Patient navigation programs

Overview of Patient Navigation Programs and Their Impact on Screening Rates

Patient navigation programs are an effective approach to reduce disparities in breast cancer screening. These programs provide personalized assistance and support to individuals throughout the screening process, guiding them from awareness to action. Patient navigators are trained professionals or lay individuals who help patients navigate the healthcare system, overcome barriers, and ensure timely access to screening services [51].

Research has shown that patient navigation programs can significantly improve screening rates among underserved populations. Navigators advocate for patients, addressing their concerns, providing information, and facilitating communication with healthcare providers. By offering culturally competent and patient-centered care, patient navigation programs can enhance engagement, decrease missed appointments, and ultimately increase the uptake of breast cancer screening [52].

Addressing Barriers in Screening through Patient Navigation

Patient navigation programs address a range of barriers that hinder individuals from accessing and completing breast cancer screening. Some common barriers include a lack of knowledge or awareness about screening, financial constraints, transportation difficulties, fear or anxiety about the screening process, and language or cultural barriers [53].

Patient navigators play a crucial role in addressing these barriers. They provide education and information about the importance of screening, addressing misconceptions and fears. Navigators also help individuals navigate the complex healthcare system by assisting with scheduling appointments, coordinating transportation, and connecting patients to financial assistance programs when needed.

Patient navigators employ a patient-centered approach, considering individual preferences, cultural beliefs, and language needs. They ensure individuals have the necessary support and resources to overcome barriers and complete the screening process. Patient navigation programs help bridge the gap between awareness and action by offering personalized guidance and support, leading to increased screening rates [27].

Success Stories and Challenges Faced by Patient Navigation Programs

Patient navigation programs have successfully improved breast cancer screening rates and reduced disparities. Numerous studies have shown that these programs have increased screening uptake, particularly among underserved populations, including racial and ethnic minorities, low-income individuals, and those with limited access to healthcare services [54,55].

Success stories highlight the impact of patient navigation programs in reducing disparities. For example, in certain communities, patient navigation programs have significantly improved screening rates, earlier detection of breast cancer, and improved access to timely follow-up care. By addressing barriers and providing personalized support, patient navigation programs have successfully empowered individuals to take action and prioritize their breast health [54,55].

However, patient navigation programs also face challenges. Limited funding, workforce shortages, and the need for ongoing training and support for patient navigators are common obstacles. Coordinating care among healthcare providers and ensuring effective communication can also be challenging. Moreover, the sustainability and scalability of patient navigation programs remain important considerations to ensure long-term impact and reach [54,55]. Despite these challenges, patient navigation programs remain crucial in reducing disparities in breast cancer screening. Their success stories and positive impact highlight the value of personalized support and assistance in guiding individuals from awareness to action [54,55].

Evaluation of efforts and limitations

Critical Evaluation of the Effectiveness of Various Interventions

It is essential to critically evaluate the effectiveness of the interventions discussed in this review article. While each intervention has shown promise in reducing disparities in breast cancer screening, their impact can vary based on contextual factors and the specific populations being targeted [56].

Policy and legislation changes have improved access to screening services, particularly for underserved populations. However, the effectiveness of these changes depends on the implementation and enforcement of the policies and the availability of resources to support their implementation [57].

Community-based programs have demonstrated success in increasing screening rates among underserved populations. Their localized approach, partnerships, and tailored strategies have effectively addressed barriers and improved awareness, education, and access to screening services. However, the scalability and sustainability of these programs can be challenging, and there is a need for continued funding and support [58].

Culturally competent interventions have been effective in engaging diverse populations and reducing disparities. By addressing cultural barriers and providing tailored messaging and language support, these interventions have improved awareness and participation in screening. However, cultural diversity within populations requires ongoing efforts to ensure that interventions are inclusive and responsive to the needs of various cultural groups [59].

Technological advancements have expanded access to screening services through telemedicine, mobile health applications, and digital tools. These innovations have shown promise in increasing convenience and engagement, particularly for individuals with limited mobility or residing in underserved areas. However, barriers, such as limited digital literacy and access to technology, need to be addressed to ensure equitable implementation [60]. Patient navigation programs have significantly impacted screening rates by addressing barriers and providing personalized support. These programs have successfully improved access, reduced missed appointments, and increased screening uptake. However, challenges, such as limited resources, workforce shortages, and sustainability, remain important considerations.

Identification of Challenges and Limitations Faced by These Efforts

Limited Resources: Many interventions require adequate funding, infrastructure, and trained personnel to be effective. Limited resources can hinder the implementation and sustainability of interventions, particularly in underserved areas with limited healthcare infrastructure [61].

Health Disparities: Addressing disparities in breast cancer screening also involves addressing underlying social determinants of health, such as socioeconomic status, education, and access to healthcare. Interventions focusing solely on screening may not fully address these broader systemic issues [62].

Cultural Sensitivity: Developing culturally competent interventions requires understanding diverse cultural backgrounds, beliefs, and practices. It can be challenging to ensure that interventions effectively address the needs of all cultural groups within a population [63].

Access and Equity: While interventions aim to improve access to screening services, there are still challenges related to transportation, geographic location, and insurance coverage that must be addressed to ensure equitable access for all populations [64].

Lessons Learned and Areas for Improvement

The efforts to reduce disparities in breast cancer screening have provided valuable lessons and insights. Some key lessons learned include the following:

Collaborative Approaches: Collaboration among healthcare providers, community organizations, policymakers, and other stakeholders is critical for the success of interventions. By leveraging the strengths and expertise of different partners, interventions can be more comprehensive and effective [65].

Tailored Approaches: Tailoring interventions to the target populations' specific needs, preferences, and cultural contexts enhances their relevance and effectiveness. This requires ongoing community engagement, cultural sensitivity, and the inclusion of diverse voices in program design and implementation [66].

Multilevel Interventions: Disparities in breast cancer screening are influenced by individual, community, and systemic factors. Interventions should address barriers at multiple levels, combining individual-focused strategies with community-based programs and policy changes [67].

Evaluation and Continuous Improvement: Ongoing evaluation and monitoring of interventions are crucial to understand their impact and identify areas for improvement. This includes collecting data on screening rates, patient experiences, and the effectiveness of specific components of interventions [68].

Future directions and recommendations

Gaps in the Existing Literature on Reducing Disparities in Breast Cancer Screening

Long-Term Outcomes: More research is needed to examine the long-term outcomes of interventions to reduce disparities in breast cancer screening. This includes evaluating the impact of interventions on cancer stage at diagnosis, survival rates, and overall health outcomes [69].

Intersectionality: Intersectionality, which considers how multiple social identities intersect and impact health outcomes, is an important aspect to explore. Further research is needed to understand the unique challenges faced by individuals who belong to multiple marginalized groups and how interventions can be tailored to address their specific needs [70].

Cost-Effectiveness: Assessing the cost-effectiveness of interventions is crucial for sustainability and scalability. Future research should focus on evaluating the economic impact of interventions, including cost savings associated with early detection and reduced cancer treatment costs [71].

Proposed Research Directions for Further Addressing Disparities

Implementation Science: Research should focus on understanding the factors that influence the successful implementation of interventions. This includes exploring barriers and facilitators at the organizational, provider, and patient levels to inform effective implementation strategies [72].

Health System Changes: Investigating the impact of health system changes, such as improved reimbursement policies, increased access to primary care, and integrated care models, on reducing disparities in breast cancer screening can provide insights into broader systemic approaches to address disparities [73].

Health Literacy and Education: Research should examine the role of health literacy and educational interventions in improving knowledge, awareness, and decision-making regarding breast cancer screening. This includes evaluating the effectiveness of different educational approaches and interventions tailored to diverse populations [74].

Recommendations for Policymakers, Healthcare Providers, and Researchers

Policymakers: Policymakers should prioritize equity in healthcare and allocate resources to support interventions to reduce disparities. This includes advocating for policies that improve access to screening services, provide financial assistance for underserved populations, and promote culturally competent care [75].

Healthcare Providers: Healthcare providers should implement culturally sensitive and patient-centered approaches. This includes addressing language barriers, offering educational materials in multiple languages, and providing culturally appropriate care. Collaboration with community organizations and patient navigators can enhance access and support for underserved populations [76].

Researchers: Researchers should prioritize research on interventions that target specific populations at higher risk of disparities, including racial and ethnic minorities, individuals with low socioeconomic status, and those in underserved areas. In addition, interdisciplinary collaboration and mixed-methods approaches can provide comprehensive insights into the complex factors influencing disparities in breast cancer screening [77]. By implementing these recommendations, policymakers, healthcare providers, and researchers can contribute to reducing disparities in breast cancer screening and promoting equitable access to care.

## Conclusions

Reducing disparities in breast cancer screening requires a comprehensive and multidimensional approach that addresses barriers at various levels. This review article has examined several key interventions to bridge the gap between awareness and action in breast cancer screening. The reviewed interventions, including policy and legislation changes, community-based programs, culturally competent interventions, technological advancements, and patient navigation programs, have shown promise in improving screening rates and reducing disparities among underserved populations. These interventions have successfully addressed barriers, such as lack of awareness, financial constraints, cultural barriers, and limited access to healthcare services. However, challenges and limitations exist, including limited resources, health disparities, cultural sensitivity, and access and equity issues. Evaluating these interventions' effectiveness and identifying improvement areas is crucial. Lessons learned from these interventions highlight the importance of collaborative approaches, tailored strategies, and multilevel interventions.
